# CT-based radiogenomic prediction of ICAM1 and RAET1E as biomarkers of NK cytotoxicity in clear cell renal cell carcinoma

**DOI:** 10.3389/fimmu.2026.1773251

**Published:** 2026-06-03

**Authors:** Xinwei Ma, Jiao Yang, Xusheng Qian, Xin Dou, Shiliang Ji, Yakang Dai, Yi Yang, Yi Wang, Jianbing Zhu

**Affiliations:** 1 Department of Radiology, Suzhou Hospital, Affiliated Hospital of Medical School, Nanjing University, Suzhou, China; 2 Suzhou Key Laboratory for Integrated Stroke Prevention, Treatment, and Rehabilitation, Suzhou Hospital, Affiliated Hospital of Medical School, Nanjing University, Suzhou, China; 3 Suzhou Research Center of Medical School, Suzhou Hospital, Affiliated Hospital of Medical School, Nanjing University, Suzhou, China; 4 Suzhou Institute of Biomedical Engineering and Technology, Chinese Academy of Sciences, Suzhou, China; 5 Department of Radiology, the Second Affiliated Hospital of Soochow University, Suzhou, Jiangsu, China; 6 Department of Nuclear Medicine, the Second Affiliated Hospital of Soochow University, Suzhou, Jiangsu, China

**Keywords:** ccRCC, ICAM1, natural killer cell, radiogenomics, RAET1E

## Abstract

**Introduction:**

This study aims to develop a noninvasive CT-based radiogenomic framework to estimate imaging-associated biomarkers related to natural killer (NK) cell cytotoxicity in clear cell renal cell carcinoma (ccRCC). By linking imaging features to underlying molecular pathways, we address the limitations of invasive tissue sampling in assessing tumor heterogeneity and immune microenvironment.

**Methods:**

This study analyzed preoperative contrast-enhanced CT images from 143 patients with histologically confirmed ccRCC and transcriptomic data from 538 TCGA-KIRC tumor samples. Radiomic features (3,176 per tumor) were extracted using PyRadiomics from manually segmented 3D tumor volumes (validated by two radiologists), spanning seven feature classes and eight image filters. Feature selection included variance filtering, the Mann-Whitney U test for gene-expression stratification (high/low groups), and redundancy removal via Pearson correlation analysis (|r| > 0.9), with AUC prioritization. An *L_1_*-penalized support vector machine model with leave-one-out cross-validation was developed to estimate expression levels of ICAM1 and RAET1E, two NK cytotoxicity-related biomarkers. External validation was performed using tissue microarrays and immunohistochemistry from 26 independent ccRCC cases. Differential gene expression was assessed using edgeR (FDR < 0.05, fold-change > 2) and survival analysis via Cox regression.

**Results:**

Transcriptomic analysis identified 835 imaging-associated genes enriched in immune-related pathways, with significant representation of the NK cell-mediated cytotoxicity pathway (KEGG, *P* < 0.05). Among candidate genes, ICAM1 and RAET1E demonstrated the strongest radiogenomic associations (AUC = 75.7% and 67.3%, respectively). Immunohistochemistry confirmed increased ICAM1 and decreased RAET1E expression in ccRCC tissues. Higher ICAM1 and lower RAET1E expression were associated with advanced tumor stage and unfavorable survival outcomes. External validation of the *L_1_*-SVM model achieved predictive accuracies of 76.92% for ICAM1 and 73.08% for RAET1E, supporting the preliminary feasibility of this radiogenomic approach.

**Conclusions:**

Our findings suggest that CT-derived radiomic features may provide noninvasive imaging correlates of biomarkers related to NK cytotoxicity-in ccRCC. Radiogenomic analysis of ICAM1 and RAET1E may provide a complementary exploratory framework for noninvasive immune characterization and biomarker research in ccRCC.

## Introduction

Renal cell carcinoma (RCC) is the most common type of kidney cancer in adults, with clear cell renal cell carcinoma (ccRCC) representing the most prevalent histological subtype, followed by papillary RCC and chromophobe RCC. ccRCC accounts for the majority of RCC-related deaths and is recognized as one of the most immunologically distinct tumor types, characterized by abundant intratumoral immune cell infiltration (1, 2) and a complex immune landscape associated with clinically meaningful responses to PD-1/PD-*L_1_*-targeting immunotherapies (3, 4). Emerging evidence also suggests that renal malignancy may share broader patterns of immune dysfunction, inflammatory signaling, and molecular pathway dysregulation observed in other kidney diseases (5, 6). Over the past decade, emerging technologies such as proteomics, single-cell RNA sequencing, immunohistochemistry, and mass cytometry have revealed the complexity and diversity of immune infiltration in ccRCC. These immune cells exhibit distinct differentiation and activation states, contributing to tumor heterogeneity and influencing both prognosis and therapeutic response (7–9). Such immune complexity is increasingly recognized as a key determinant of treatment resistance and clinical outcomes in renal malignancy. However, characterizing tumor subtypes and immune infiltration patterns currently depends on invasive tissue sampling, typically obtained postoperatively.

Computed tomography (CT) is routinely used to diagnose RCC and captures the macroscopic characteristics of the entire tumor volume. Radiogenomics, which integrates quantitative imaging phenotypes with genomic data, offers a potentially useful noninvasive approach for characterizing tumor heterogeneity and has shown increasing promise in renal cancer management (10–12). Recent studies have shown that radiomic signatures derived from CT imaging may serve as noninvasive biomarkers for identifying ccRCC molecular subtypes and key driver mutations such as VHL, BAP1, and PBRM1 (13–16). Building on this, we hypothesized that CT-based imaging phenotypes may also provide noninvasive imaging correlates of immune-related molecular biomarkers in ccRCC. Specifically, we identified differentially expressed genes in ccRCC and analyzed their correlation with radiomic features extracted from contrast-enhanced CT images.

Pathway enrichment analysis showed that imaging-associated genes were significantly enriched in pathways related to natural killer cell-mediated cytotoxicity, from which ICAM1 and RAET1E were selected as candidate biomarkers for subsequent radiogenomic modeling.

## Materials and methods

### Study population

Differential gene expression analysis was performed using RNA sequencing data from 538 ccRCC tumor samples and 72 normal kidney tissues obtained from The Cancer Genome Atlas (TCGA-KIRC) dataset. Preoperative CT images were also collected using the following inclusion criteria: 1) histopathologically confirmed ccRCC after surgical resection, and 2) availability of contrast-enhanced CT (CECT) scans in the corticomedullary phase (CP) prior to surgery or radiotherapy. Exclusion criteria included: 1) availability of only MR images; 2) availability of only non-contrast CT scans; 3) history of surgery and/or chemotherapy prior to imaging; and 4) presence of multiple lesions. After applying these criteria, CT images from 143 patients were identified for radiomic feature extraction and further analysis. Patient characteristics are shown in **Table 1**.

**Table 1:** Patient characteristics of ccRCC.

Characteristic	Low stage(n=58)	High stage(n=85)	*P value*
Sex(n,%)			0.078
Male	29(20.28%)	64(44.75%)	
Female	29(20.28%)	21(14.69%)	
age(year)	57.05 ± 11.85	60.65 ± 12.04	0.920
Tumor size(mm)	59.20 ± 33.26	63.69 ± 29.98	0.772

*P* value<0.05 the difference is statistically significant.

In addition, immunohistochemical (IHC) analysis was performed on tissue samples from 60 ccRCC patients who underwent nephrectomy at the Suzhou Hospital, Affiliated Hospital of Medical School, Nanjing University between October 2020 and December 2023. Inclusion criteria for the IHC cohort were: 1) availability of preoperative CECT scans performed using a standard three-phase renal mass CT imaging protocol [CP, nephrographic phase (NP), delayed phase (DP)]; and 2) postoperative histopathologic confirmation of ccRCC. Exclusion criteria included: 1) incomplete tumor coverage on CT images; and 2) suboptimal CT imaging quality. Based on these criteria, 26 cases were included for IHC analysis.

All procedures involving human participants were conducted in accordance with the ethical standards of the institutional research committee and the Declaration of Helsinki (as revised in 2013). This study was approved by the Institutional Review Board of Suzhou Science and Technology City Hospital (the Suzhou Hospital, Affiliated Hospital of Medical School, Nanjing University) (IRB202202003RI).

### Radiomic feature extraction

Primary tumor segmentation was performed by a radiologist with 8 years of experience using an in-house developed artificial intelligence-assisted diagnosis software (AIMS) (17), and independently reviewed by a senior radiologist with 27 years of experience to ensure accuracy and consistency. The entire tumor volume of interest (VOI) was manually delineated on each axial slice of the CP CT images. The VOI included all visible tumor components, such as necrotic, cystic, and hemorrhagic areas, while excluding normal renal tissue, perinephric fat, and sinus fat.

**Figure 1:**
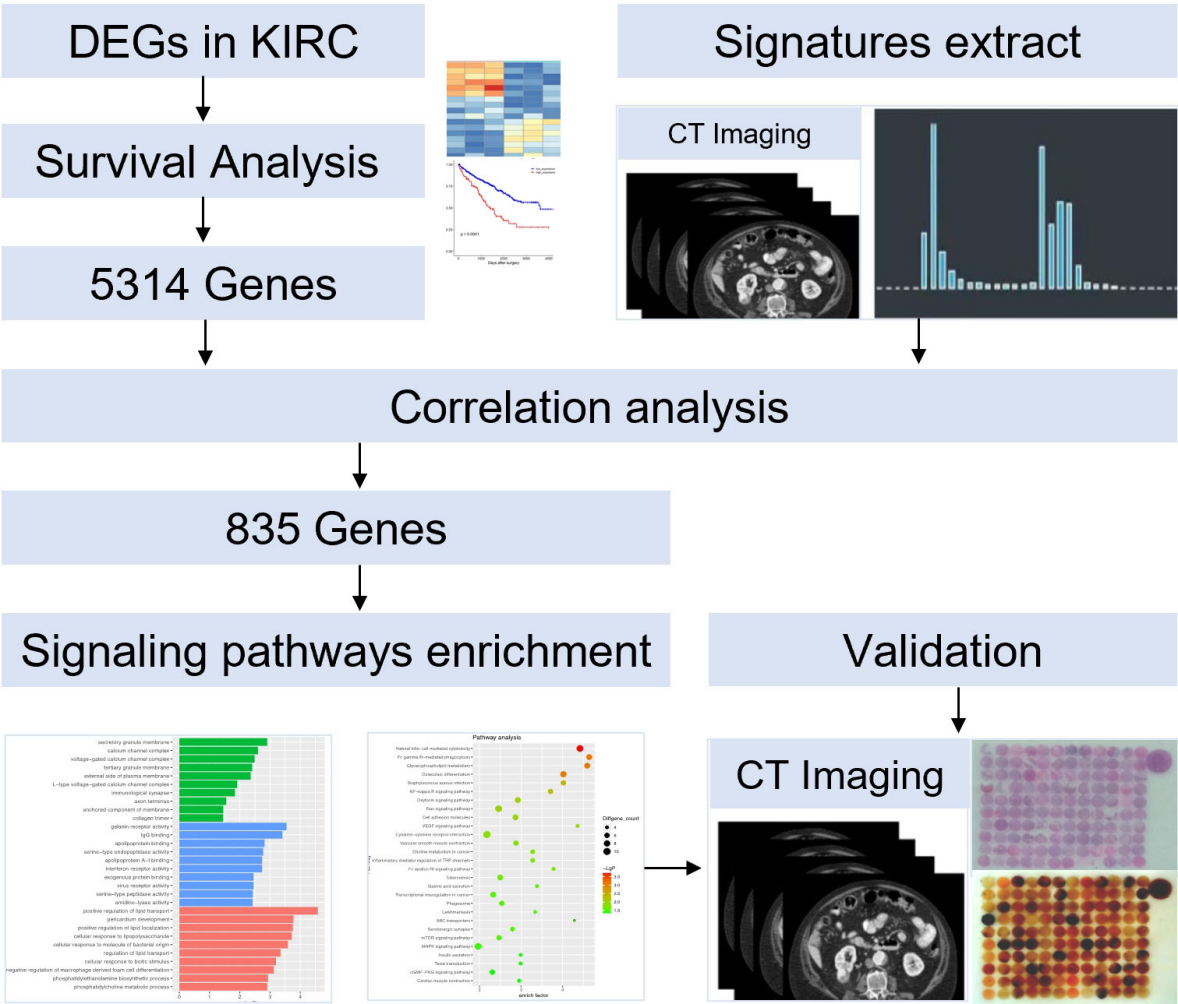
Workflow of the radiogenomic analysis pipeline in ccRCC. Differentially expressed genes (DEGs) from TCGA-KIRC were identified and screened through survival analysis and imaging-transcriptomic matching. Radiomic features were extracted from contrast-enhanced CT images and integrated with transcriptomic profiles for correlation analysis. Genes showing significant radiogenomic associations were prioritized for pathway enrichment analysis. Candidate biomarkers identified from NK-cell-related pathways were subsequently evaluated and validated using an independent tissue microarray and imaging cohort.

All VOIs were resampled to isotropic voxel spacing of 1×1×1 mm using B-spline interpolation. To ensure all voxel intensity values were positive, a constant value of 1000 was added to each voxel. A fixed bin width of 25 was used for high-order feature extraction. Radiomic features were extracted using the AIMS software, which integrates the open-source PyRadiomics package, covering seven feature groups: (I) first-order features; (II) shape-based features; (III) gray-level co-occurrence matrix (GLCM) features; (IV) gray-level run length matrix (GLRLM) features; (V) gray-level size zone matrix (GLSZM) features; (VI) neighboring gray-tone difference matrix (NGTDM) features; and (VII) gray-level dependence matrix (GLDM) features. The overall radiogenomic workflow is illustrated in **Figure 1**.

To capture multi-scale and texture-enhanced information, eight image filters were applied to the original CT images to generate derived image sets, including Laplacian of Gaussian (LoG), wavelet, logarithm, square, square root, exponential, gradient, and 3D local binary pattern (LBP). The radiomic features were then extracted from both the original and derived images. In total, 3,176 radiomic features per tumor were used for subsequent analysis and model development.

To assess the reproducibility of radiomic feature extraction, a subset of cases was independently segmented by a second radiologist, and intraclass correlation coefficients (ICCs) were calculated to evaluate interobserver agreement. Radiomic features with ICC values greater than 0.75 were considered reproducible and were retained for subsequent analysis.

### Feature selection

As a preprocessing step, all radiomic features were scaled to the range [-1, 1] to ensure that features with larger numeric ranges did not dominate those with smaller ranges, and to avoid numerical instability during computation. To identify a discriminative subset of radiomic features for predicting the expression level of specific genes, a multi-step feature selection procedure was performed:

1. Low variance filtering: Features with low variance (< 0.05) were removed as they provide limited discriminatory power.
2. Univariate statistical testing. The remaining features were subjected to the Mann–Whitney U test to compare distributions between high-expression and low-expression groups defined by gene expression stratification. Features with *P* values greater than 0.05 were considered statistically non-significant and were excluded from further analysis.
3. Redundancy reduction: Pairwise Pearson correlation coefficients were calculated to identify and remove redundant features. When two features exhibited high correlation (|r| > 0.9), their predictive performance was compared using the area under the receiver operating characteristic curve (AUROC), and the feature with the higher AUROC value was retained.

### Radiogenomic model construction

To reduce the risk of overfitting and selection bias, we employed the support vector machine (SVM) with *L*_1_-norm regularization for radiogenomic model construction. This *L*_1_-penalized SVM not only enables robust feature selection but also performs automatic feature selection by promoting sparsity in the model coefficients (18). Model performance was evaluated using leave-one-out cross-validation, which eliminates the randomness introduced by arbitrary train-test splits and provides an unbiased assessment, particularly in studies with limited sample sizes.

### Tissue microarray

Tissue microarrays were constructed using resected ccRCC samples. The samples were formalin-fixed and paraffin-embedded. The corresponding tumor regions on the donor paraffin blocks were selected according to the markings on hematoxylin and eosin (H&E)-stained sections. Cylindrical tissue cores with a diameter of 2.0 mm were obtained using a tissue microarray (TMA) instrument and transferred into recipient paraffin blocks in a systematic top-to-bottom and left-to-right order. A total of 52 ccRCC tissue cores from 26 patients were included in the array block. Five-micrometer-thick sections were cut from the array block, and H&E staining was performed to confirm the presence and quality of tumor tissue.

### IHC staining

The expression levels of ICAM1 (Intercellular Adhesion Molecule 1) and RAET1E (Retinoic Acid Early Transcript 1E) in TMA sections were assessed using immunohistochemistry (IHC). Briefly, tissue sections were dewaxed in xylene and rehydrated through graded ethanol solutions.

Tissue sections were heated in citrate buffer for 15 min in a microwave oven. After natural cooling, the sections were treated with 3% H_2_O_2_ for 10 min to block endogenous peroxidase activity. Tissue sections were incubated overnight at 4 °C with anti-N2DL4 monoclonal antibody (RAET1E-encoded protein; MA5-24328, Invitrogen; 1:80) or anti-ICAM1 monoclonal antibody (GB14040, Servicebio; 1:200). Staining for ICAM1 and N2DL4 was carried out using the Envision kit (Dako; EnVision Detection Systems). After washing, sections were incubated with secondary antibody and visualized using 3,3’-diaminobenzidine (DAB). Finally, tissue sections were counterstained with hematoxylin, dehydrated through graded alcohol, cleared in xylene, and mounted with permanent mounting medium.

Immunoreactive score (IRS) was calculated as IRS = SI × PP, where SI represents staining intensity and PP represents the proportion of positive cells. SI was graded as follows: 0, no staining; 1, weak staining; 2, moderate staining; 3, strong staining. PP was graded as follows: 0, 0–5%; 1, 6–25%; 2, 26–50%; 3, 51–75%; and 4, >75%.

### Transcriptomic-radiomic candidate gene selection

To identify imaging-associated candidate genes for downstream biological analysis, a sequential filtering framework was applied. First, differentially expressed genes between ccRCC tumor tissues and adjacent normal tissues were identified using edgeR. Second, only genes with available matched transcriptomic data from patients who also had corresponding preoperative CT imaging were retained for radiogenomic correlation analysis. Third, genes demonstrating at least one statistically significant correlation with extracted radiomic features after Benjamini–Hochberg false discovery rate (FDR) correction were classified as imaging-associated genes. Finally, to prioritize genes showing broader and more stable radiogenomic relationships while reducing isolated weak associations, genes correlated with ≥10 radiomic features were retained for pathway enrichment analysis. This threshold was used as a heuristic prioritization criterion in this exploratory study and was informed by previously published multistep radiogenomic workflows (19).

### Additional transcriptomic immune-context analysis

To further evaluate the immune relevance of the radiogenomic target genes, additional transcriptomic analyses were performed using TCGA-KIRC bulk RNA-seq data. Gene expression values were transformed as log2(TPM + 1). Immune-cell abundance was inferred using MCP-counter with HUGO gene symbols as input. The inferred NK-cell abundance score and selected immune/stromal cell populations, including cytotoxic lymphocytes, CD8 T cells, T cells, monocytic lineage cells, and endothelial cells, were used for downstream analyses. Patients were divided into high- and low-expression groups according to the median expression of ICAM1 or RAET1E, respectively. To explore their combined biological relevance, TCGA-KIRC tumors were further stratified into four groups according to median ICAM1 and RAET1E expression: ICAM1-low/RAET1E-low, ICAM1-high/RAET1E-low, ICAM1-low/RAET1E-high, and ICAM1-high/RAET1E-high. Associations with pathological stage and overall survival were additionally evaluated. All analyses were conducted in R.

### Statistical analysis

Differential expression analysis was performed using the edgeR package in R. Genes were considered significantly differentially expressed if they met the following criteria: *P* value < 0.05, false discovery rate (FDR) < 0.05, and fold change > 2 or < 0.5.

Continuous variables were compared using the Mann–Whitney U test, Wilcoxon rank-sum test, or Kruskal–Wallis test as appropriate. Correlation analyses were performed using Pearson or Spearman correlation coefficients where applicable. For radiomic feature-gene correlation analyses, *P* values were adjusted using the Benjamini–Hochberg FDR correction, and genes with at least one significant correlation after FDR correction were classified as imaging-associated genes. Categorical variables were compared using the chi-square test or Fisher’s exact test.

Overall survival was evaluated using Kaplan–Meier analysis with the log-rank test. Cox proportional hazards regression models were used to estimate hazard ratios (HRs) and 95% confidence intervals (CIs). All statistical analyses were conducted in R, and two-sided *P*-values < 0.05 were considered statistically significant unless otherwise specified.

## Results

### TCGA database analysis

A total of 26,480 differentially expressed genes were identified, among which 22,627 were upregulated and 3,853 were downregulated in tumor tissue. Of the 610 TCGA cases, 143 had corresponding preoperative CT images and were included for radiomic correlation analysis. From the transcriptomic dataset, 5,314 genes with available matched imaging-expression data were included for radiogenomic correlation analysis.

Among these, 2,751 genes demonstrated at least one statistically significant association with radiomic features after Benjamini–Hochberg FDR correction. To prioritize more robust imaging-associated signals for downstream biological interpretation, genes correlated with ≥10 radiomic features were retained to prioritize genes with broader and more stable radiogenomic associations, yielding 835 genes for pathway enrichment analysis. Patient clinical characteristics of the 143 imaging-available cases showed no statistically significant differences across key baseline variables (**Table 1**).

**Figure 2:**
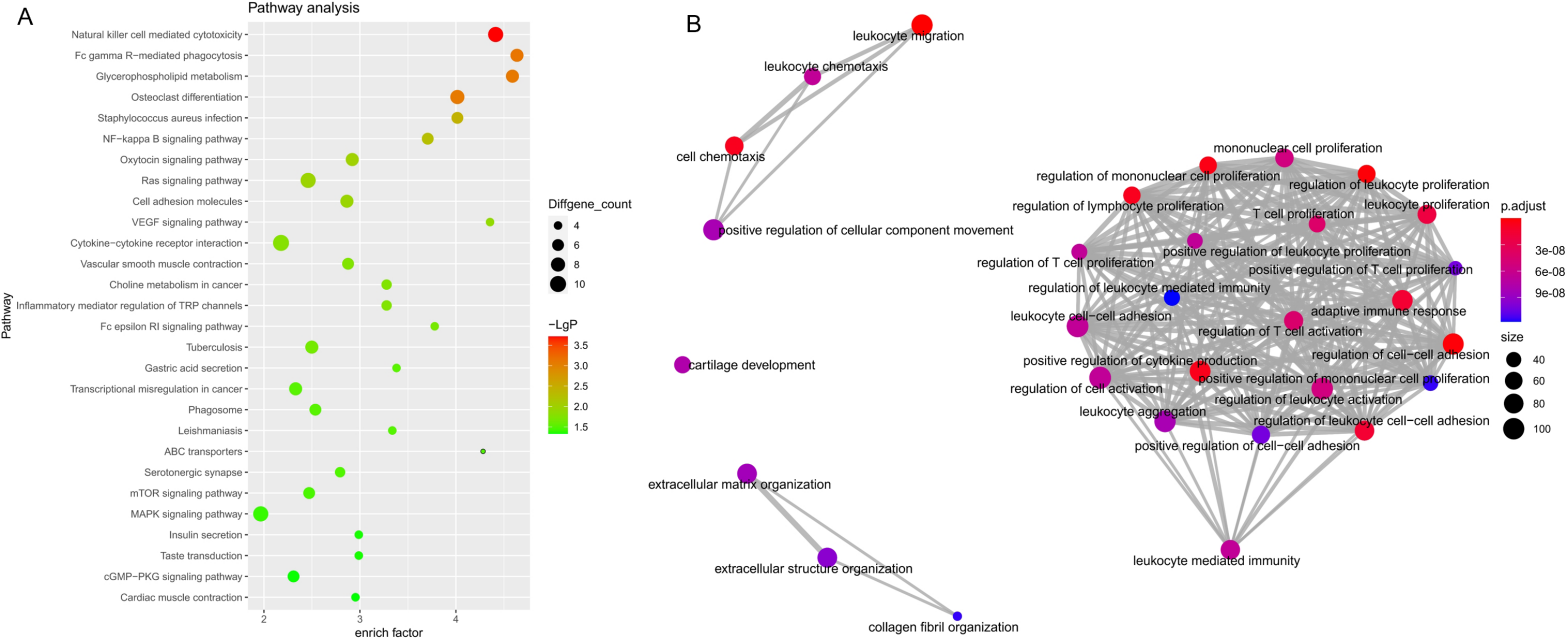
Functional enrichment analysis of imaging-associated genes in ccRCC. **(A)** KEGG pathway enrichment analysis showing significantly enriched pathways among imaging-associated genes. The natural killer cell-mediated cytotoxicity pathway was identified among the significantly enriched immune-related pathways.**(B)** Gene Ontology biological process enrichment network illustrating relationships among significantly enriched immune-associated processes, including leukocyte migration, T-cell proliferation, immune activation, and leukocyte-mediated immunity.

KEGG pathway enrichment analysis demonstrated that imaging-associated genes were significantly enriched in immune-related pathways, with notable enrichment of the natural killer cell-mediated cytotoxicity pathway (**Figure 2A**). Gene Ontology enrichment network analysis further demonstrated that these imaging-associated genes were functionally linked to immune-related biological processes, including leukocyte migration, immune activation, and regulation of lymphocyte proliferation (**Figure 2B**).

Within this pathway-related gene set, the two key candidate genes were ICAM1 and RAET1E, with AUROC values of 0.76 and 0.68, and accuracy values of 0.71 and 0.66, respectively (**Table 2**). ICAM1 expression was upregulated, whereas RAET1E expression was downregulated in ccRCC. The list of radiomics features selected by *L*_1-_SVM for prediction of ICAM1 and RAET1E expression levels are provided in **Supplementary Tables 1**, **2**.

**Table 2:** Differential expressed genes in ccRCC and their correlations with radiomics features.

Gene	Image number	Function	AUC	ACC
SHC2	49	Predicted to be involved in transmembrane receptor protein tyrosine kinase signaling pathway.	0.6878	0.6584
LAT	27	Linker For Activation Of T Cells Required for TCR (T-cell antigen receptor)- and pre-TCR-mediated signaling, both in mature T-cells and during their development	0.5681	0.5522
IFNAR2	23	Associates with IFNAR1 to form the plasma membrane receptor in the type I interferon signaling pathway	0.6348	0.6005
FCGR3A	20	This gene encodes a receptor for the Fc portion of immunoglobulin G, and it is involved in the removal of antigen-antibody complexes from the circulation	0.6681	0.5653
FCGR3B	20	Low affinity receptor. Binds complexed or aggregated IgG and also monomeric IgG	0.6177	0.5943
IFNGR2	20	Defects in IFNGR2 are a cause of mendelian susceptibility to mycobacterial disease (MSMD),	0.5823	0.5677
**ICAM1**	13	A cell surface glycoprotein which is typically expressed on endothelial cells and cells of the immune system. It binds to integrins of type CD11a/CD18, or CD11b/CD18 and is also exploited by Rhinovirus as a receptor.	**0.7566**	0.7138
PRKCG	13	a family of serine- and threonine-specific protein kinases that can be activated by calcium and second messenger diacylglycerol.	0.6293	0.6089
**RAET1E**	11	This protein functions as a ligand for NKG2D receptor, which is expressed on the surface of several types of immune cells, and is involved in innate and adaptive immune responses.	**0.6783**	0.6589

Bold values indicate the two candidate biomarkers with highest AUC (ICAM1 and RAET1E).

### Tissue validation

**Figure 3:**
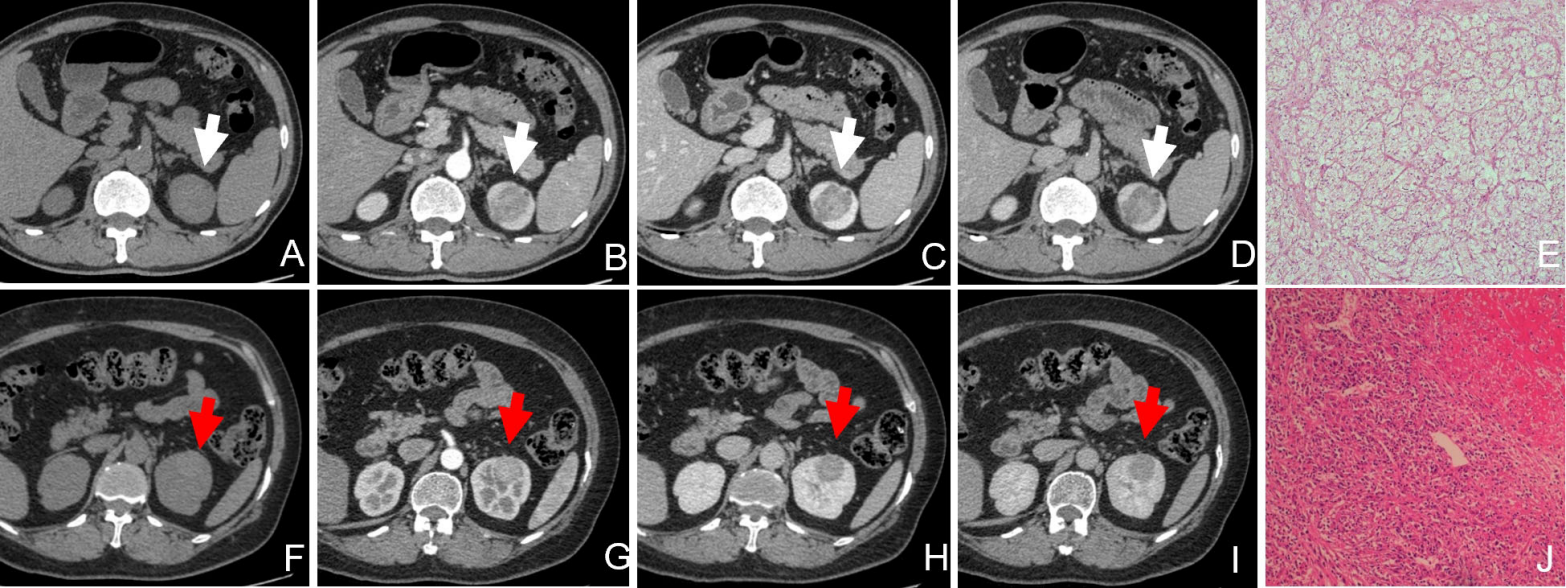
Representative CT and histopathological characteristics of low-grade and high-grade ccRCC. **(A–E)** Representative low-grade ccRCC (WHO/ISUP grade II) from a 60-year-old male. Panels **(A–D)** show unenhanced, corticomedullary, nephrographic, and excretory phase CT images, respectively. White arrows indicate the left renal tumor. Panel E shows the corresponding hematoxylin and eosin (H&E) staining. **(F–J)** Representative high-grade ccRCC (WHO/ISUP grade IV) from a 60-year-old female. Panels **(F–I)** show unenhanced, corticomedullary, nephrographic, and excretory phase CT images, respectively. Red arrows indicate the left renal tumor. Panel **(J)** shows the corresponding H&E staining. These representative cases illustrate the imaging and histopathological heterogeneity of ccRCC and provide visual context for subsequent radiogenomic analyses.

Representative imaging and histopathological characteristics of ccRCC tumors with different pathological grades are shown in **Figure 3**. Compared with WHO/ISUP grade II tumors, grade IV tumors demonstrated greater imaging heterogeneity and more pronounced pathological atypia, illustrating the substantial radiologic and histopathological heterogeneity of ccRCC.

**Figure 4:**
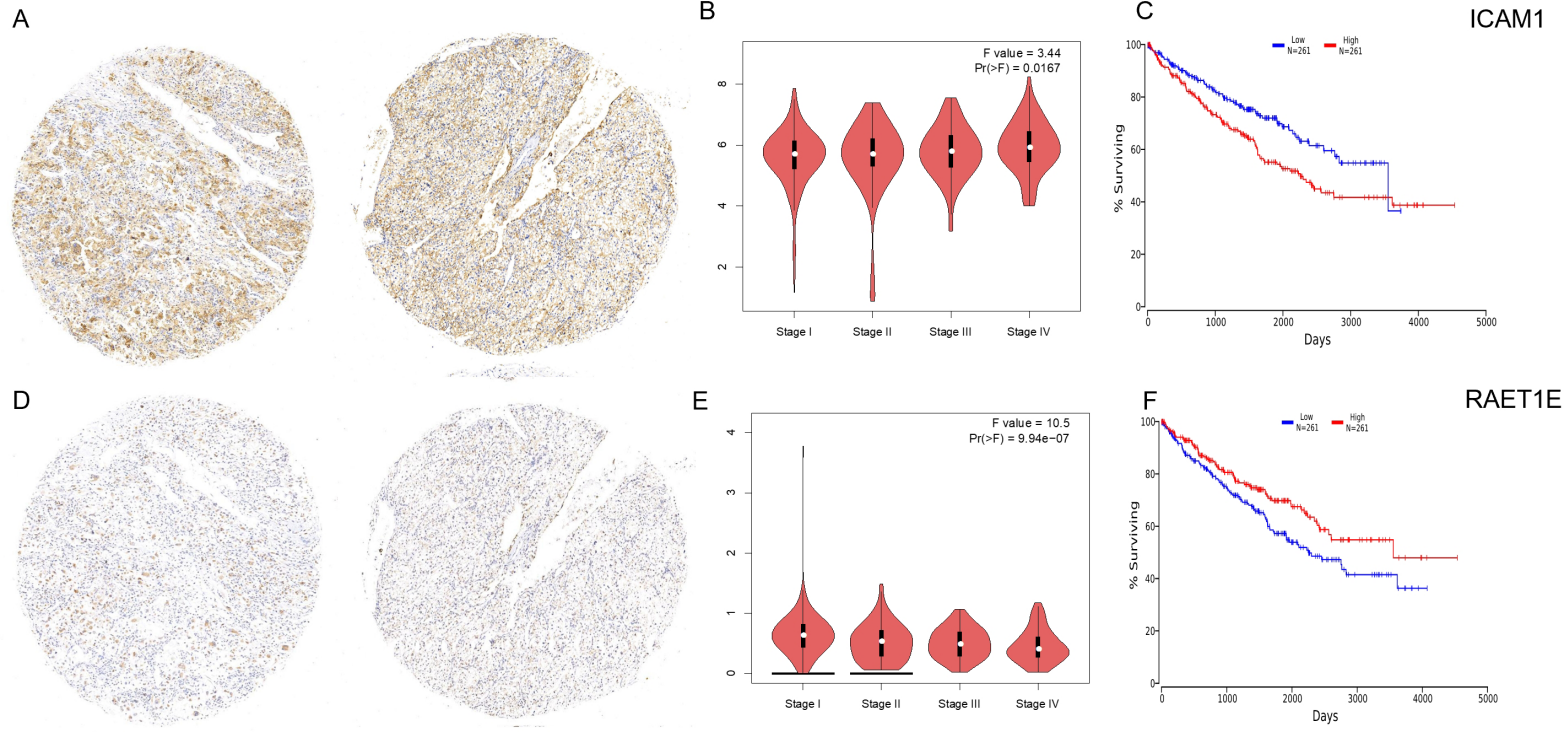
Expression patterns and prognostic associations of ICAM1 and RAET1E in ccRCC. **(A)** Representative immunohistochemical staining showing high ICAM1 expression in ccRCC tissues. **(B)** Violin plot showing the positive association between ICAM1 expression and tumor stage. **(C)** Kaplan–Meier survival analysis showing that high ICAM1 expression was associated with poorer overall survival. **(D)** Representative immunohistochemical staining showing low RAET1E expression in ccRCC tissues. **(E)** Violin plot showing the negative association between RAET1E expression and tumor stage. **(F)** Kaplan–Meier survival analysis showing that high RAET1E expression was associated with improved overall survival.

Consistent with our computational analysis, representative immunohistochemical staining demonstrated high ICAM1 expression and relatively low RAET1E expression in ccRCC tissues (**Figures 4A, D**). ICAM1 expression positively correlated with tumor stage, indicating higher expression in more advanced tumors (**Figure 4B**). In contrast, RAET1E expression showed a negative association with tumor stage (**Figure 4E**). Survival analysis revealed that high ICAM1 expression was associated with shorter overall survival (**Figure 4C**), whereas higher RAET1E expression was associated with longer survival (**Figure 4F**), suggesting prognostic associations.

**Figure 5:**
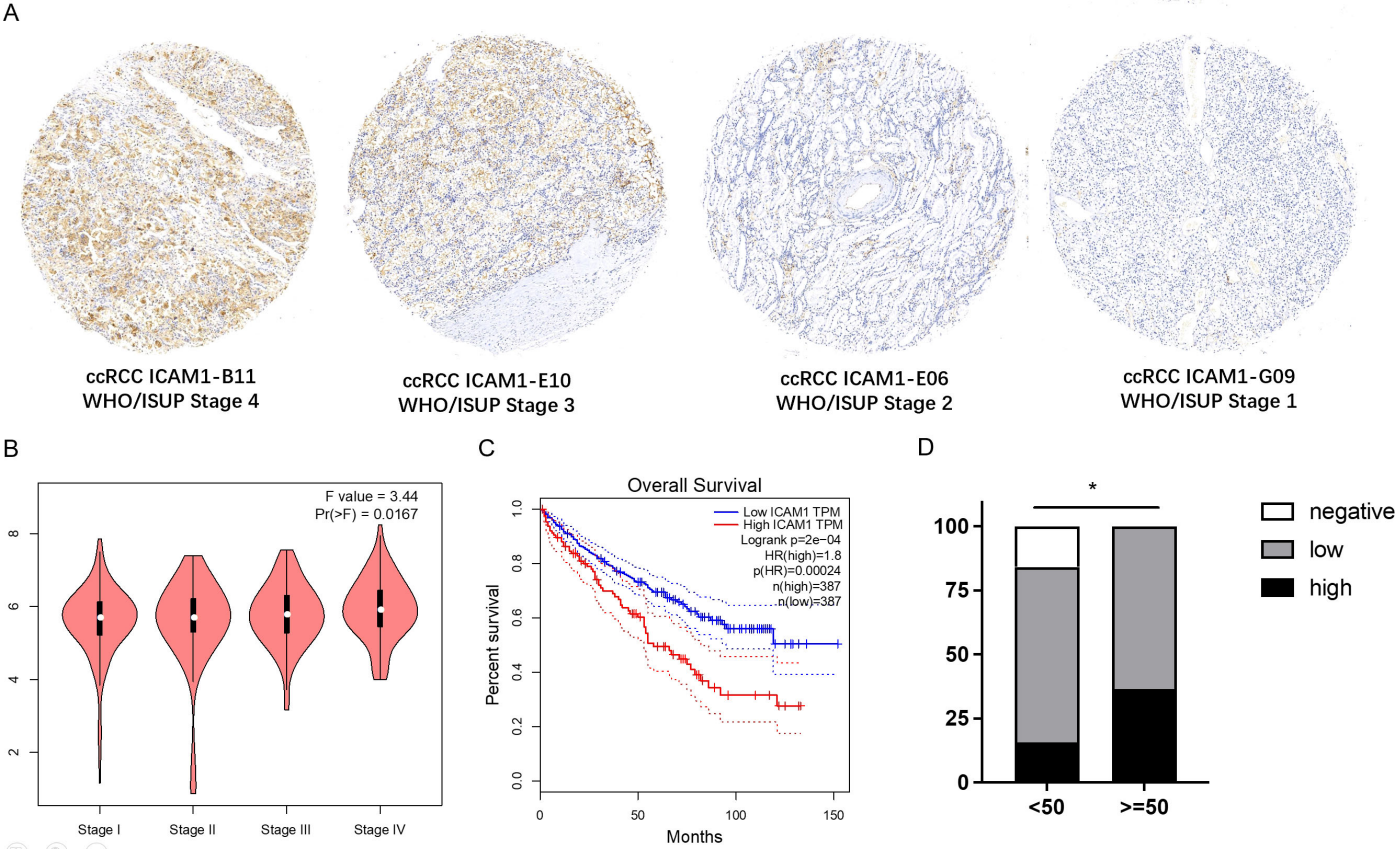
Stage-dependent expression and clinical associations of ICAM1 in ccRCC. **(A)** Representative immunohistochemical staining of ICAM1 across WHO/ISUP stages I–IV, showing progressively increased expression in advanced tumors. **(B)** Violin plot showing the positive association between ICAM1 expression and tumor stage. **(C)** Kaplan–Meier survival analysis indicating poorer overall survival in patients with high ICAM1 expression. **(D)** Distribution of ICAM1 staining categories stratified by expression (<50 and ≥50).

To further evaluate stage-dependent expression patterns, immunohistochemical staining for ICAM1 was performed in representative ccRCC samples across stages I–IV (**Figure 5A**). Representative immunohistochemical staining demonstrated progressively increased ICAM1 expression from stage I to stage IV tumors, further supporting its association with tumor progression (**Figure 5A**). Additional analyses supported associations between ICAM1 expression, tumor stage, survival outcome, and expression-category distribution (**Figures 5B–D**).

### Additional immune-context analysis

To further evaluate the immunological relevance of ICAM1 and RAET1E, MCP-counter immune deconvolution analysis was performed using TCGA-KIRC data. ICAM1 expression was significantly associated with inferred NK-cell abundance, cytotoxic lymphocytes, CD8 T cells, T cells, monocytic lineage cells, myeloid dendritic cells, neutrophils, and endothelial cells. RAET1E expression was also significantly associated with inferred NK-cell abundance and cytotoxic lymphocytes.

**Figure 6:**
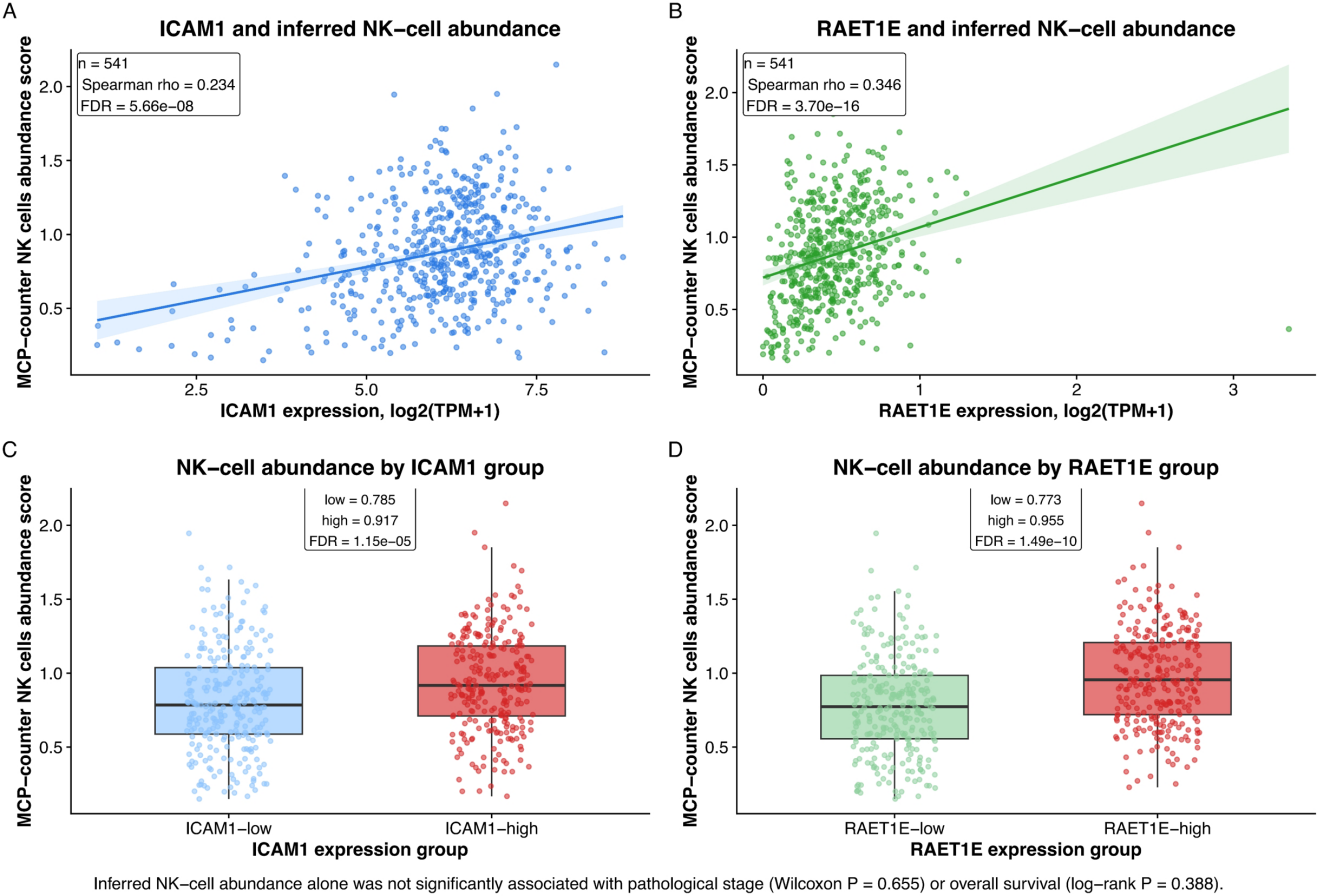
Immune deconvolution analysis of ICAM1 and RAET1E in TCGA-KIRC. **(A)** Correlation between ICAM1 expression and inferred NK-cell abundance estimated by MCP-counter. **(B)** Correlation between RAET1E expression and inferred NK-cell abundance **(C)** Comparison of NK-cell abundance between ICAM1-high and ICAM1-low tumors. **(D)** Comparison of NK-cell abundance between RAET1E-high and RAET1E-low tumors. Both ICAM1 and RAET1E expression showed significant positive associations with inferred NK-cell abundance.

ICAM1 and RAET1E expression both showed significant positive correlations with inferred NK-cell abundance (**Figures 6A, B**). In addition, tumors with high ICAM1 or high RAET1E expression exhibited significantly higher inferred NK-cell abundance than their corresponding low-expression groups (**Figures 6C, D**).

**Figure 7:**
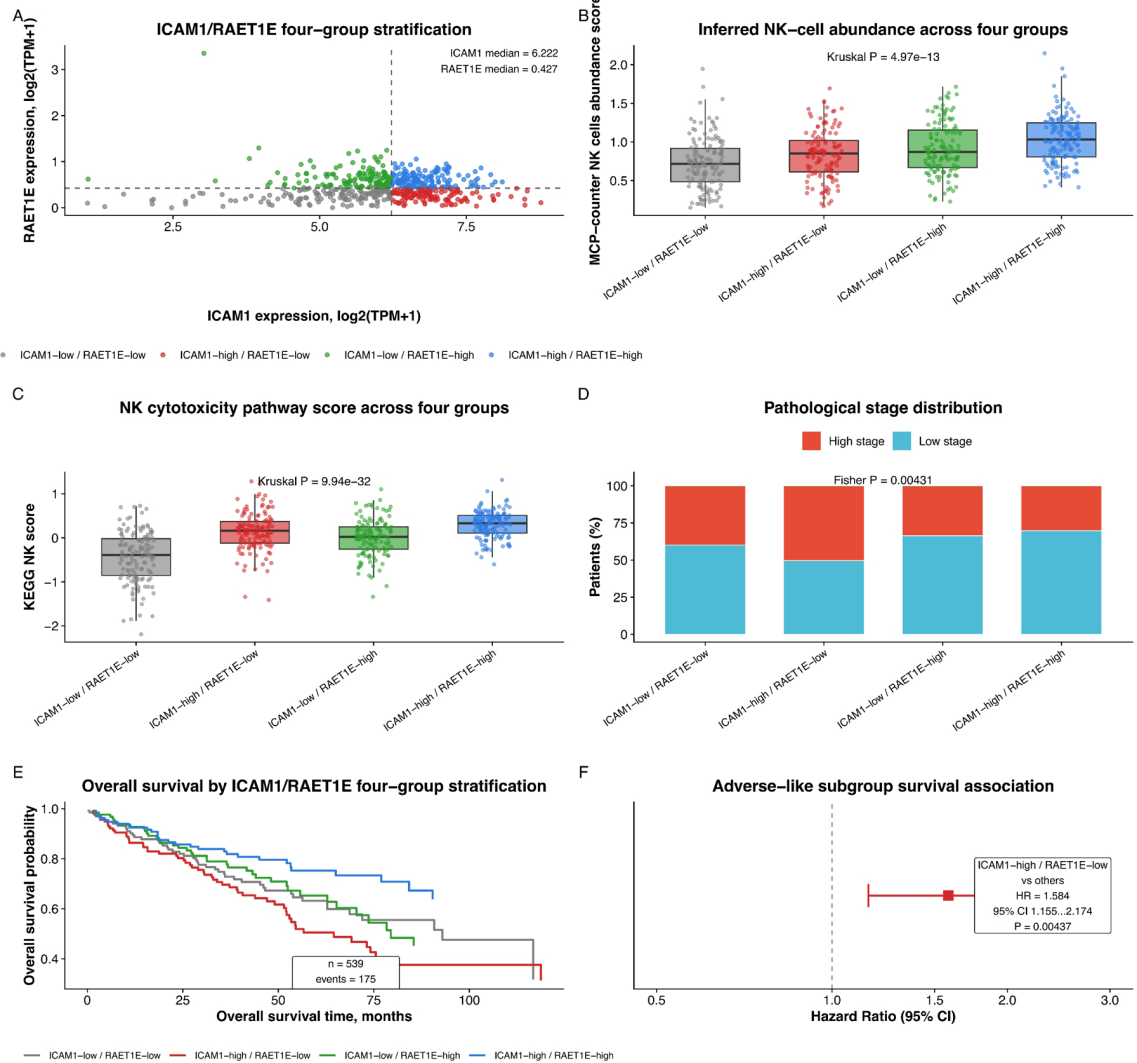
Combined ICAM1/RAET1E stratification reveals immune-context and clinical heterogeneity in TCGA-KIRC. **(A)** Four-group stratification based on median ICAM1 and RAET1E expression. **(B)** Comparison of inferred NK-cell abundance across the four groups. **(C)** Comparison of KEGG NK cytotoxicity pathway scores across the four groups. **(D)** Distribution of pathological stage across the four groups. **(E)** Kaplan–Meier survival analysis among the four subgroups. **(F)** Forest plot comparing the ICAM1-high/RAET1E-low subgroup with all remaining tumors combined. The ICAM1-high/RAET1E-low subgroup showed an unfavorable survival association relative to the remaining tumors.

TCGA-KIRC tumors were further stratified into four groups according to median ICAM1 and RAET1E expression levels (**Figure 7A**). Combined ICAM1/RAET1E stratification identified significant differences in inferred NK-cell abundance (**Figure 7B**), KEGG NK cytotoxicity pathway scores (**Figure 7C**), pathological stage distribution (**Figure 7D**), and overall survival among the four subgroups (**Figure 7E**). In particular, the ICAM1-high/RAET1E-low subgroup showed an unfavorable survival association compared with the remaining tumors (**Figure 7F**).

### Predictive model performance

Using CT data from an independent 26-patient cohort for pilot external validation, the model achieved an accuracy of 76.92%, with 100% sensitivity and 53.85% specificity for ICAM1. For RAET1E, the model achieved an accuracy of 73.08%, with 100% sensitivity and 46.15% specificity (**Table 3**). These findings suggest preliminary external applicability, although the relatively modest specificity and limited sample size require confirmation in larger multicenter cohorts.

**Table 3:** Prediction performance of ICAM1 and RAET1E expression levels based on the *L*_1-_penalized SVM.

ICAM1			RAET1E		
Accuracy	Sensitivity	Specificity	Accuracy	Sensitivity	Specificity
0.7692	1.00	0.5385	0.7308	1.00	0.4615

## Discussion

The tumor microenvironment plays a central role in ccRCC progression, metastatic behavior, and response to systemic therapies, particularly immune checkpoint inhibition (20–22). In our study, clustering analysis of differentially expressed genes revealed that a substantial proportion of aberrantly expressed genes in ccRCC were enriched in immune-regulatory pathways, most notably the natural killer cell-mediated cytotoxicity pathway. This finding is consistent with growing evidence suggesting that ccRCC is characterized by profound immune dysregulation, dynamic tumor–immune interactions, and spatially heterogeneous antitumor immunity (22, 23).

Our findings align with the emerging paradigm of radiogenomics as a noninvasive strategy for characterizing tumor biology, while also highlighting ongoing challenges and opportunities for further refinement (24). The observed association between radiomics signatures and biomarkers linked to the NK cell-mediated cytotoxicity pathway suggests a potential relationship between tumor morphology, as captured by imaging, and immune regulation.

Two genes, ICAM1 (25) and RAET1E (26, 27), emerged as key components within this pathway, with expression levels showing significant correlations with distinct radiomic features. ICAM1 is a pro-inflammatory adhesion molecule that facilitates immune cell infiltration and has been implicated in tumor progression and metastasis (25). Its upregulation in ccRCC is consistent with prior studies linking elevated ICAM1 expression to advanced tumor stage and poor prognosis, potentially through promotion of immune evasion or chronic inflammation. Conversely, RAET1E is a stress-induced ligand for NK-cell receptors. Its downregulation in ccRCC is consistent with a tumor-suppressive role, given its function in activating NK-cell responses. Stronger NKG2D signaling and higher ligand expression may promote more effective NK-cell or CD8^+^ T cell responses, leading to enhanced clearance of malignant cells. The inverse correlation between RAET1E expression and tumor stage further supports its potential role as a protective biomarker.

Mechanistic insights from Xu et al. (28) provide further context. Their study identified a dysfunctional CD49a^+^CD9^+^ NK-cell subpopulation in advanced ccRCC characterized by reduced cytotoxicity markers (GZMB, PRF1) despite preserved cytokine production. This phenotype was enriched in advanced disease (*P* = 0.00039 vs. localized ccRCC) and associated with reduced CD107a expression, a marker of NK-cell degranulation. The observed radiomic signatures may therefore reflect spatial patterns of functionally impaired NK cells within the tumor microenvironment. This interpretation is further supported by recent evidence indicating that tumor-associated molecules such as MUC1 can modulate the immune microenvironment through complement activation and regulation of immune cell infiltration, promoting an immunosuppressive phenotype characterized by increased macrophages and reduced CD8^+^ T cells (29). Prior radiogenomic studies in ccRCC have primarily focused on imaging correlates of canonical driver alterations such as VHL, PBRM1, and BAP1, whereas immune-associated radiogenomic biomarkers remain comparatively underexplored (24, 30). This shift is consistent with recent literature emphasizing tumor microenvironment heterogeneity as a critical determinant of prognosis and therapeutic response.

The enrichment of the NK cytotoxicity pathway in our dataset suggests potential immunotherapeutic relevance. Recent evidence indicates progressive NK-cell dysfunction in advanced ccRCC, characterized by impaired cytotoxicity despite preserved cytokine signaling, highlighting a possible therapeutic opportunity (28). These pathways may therefore represent candidate targets for strategies aimed at restoring cytotoxic function, including NK-engaging biologics and checkpoint modulators. Radiomic features associated with ICAM1 and RAET1E expression may also provide biological insights. For ICAM1, significant correlations with first-order features such as Root Mean Squared and 90th Percentile could reflect tumor cellularity and intralesional heterogeneity.

In addition, high-order features such as HGLRE (emphasizing high gray-level concentrations) may indicate densely cellular or highly vascularized tumor regions, consistent with previous studies suggesting that increased microvascular density and tumor-associated macrophages and mast cells contribute to angiogenesis and tumor progression in ccRCC (31). The negative correlation with Joint Energy, a homogeneity metric, suggests that ICAM1-high tumors are structurally more complex, possibly due to immune infiltration. In contrast, radiomic features linked to RAET1E expression, such as Gray Level Variance (GLSZM) and Dependence Variance (GLDM), may reflect structural disorganization in tumors with reduced NK-cell activity, potentially representing immune-suppressed microenvironments. These findings suggest a potential role for radiogenomics in providing insights into tumor–immune interactions, although further validation is required before it can be considered a predictive tool. More broadly, these findings may be interpreted within the context of renal immune dysregulation, where chronic inflammatory signaling, stromal remodeling, and innate immune dysfunction have been suggested to contribute to both malignant and non-malignant kidney diseases (10, 22, 32). At a more refined level, complex interactions between immune cell subsets, metabolic reprogramming, and signaling pathways may collectively influence the tumor microenvironment. Recent integrative analyses combining bulk and single-cell RNA sequencing have further indicated that ccRCC exhibits substantial heterogeneity in immune cell composition and functional states, with macrophages, T cells, and NK cells interacting through pathways related to inflammation, complement activation, and angiogenesis (33).

We acknowledge several limitations with this study. First, although our radiogenomic model demonstrated relatively high sensitivity in the external cohort, specificity remained modest in this exploratory analysis. This likely reflects the small validation sample size (n = 26), class imbalance, and potential overfitting inherent to exploratory single-center datasets (34). Although the *L_1_*-SVM model (18) was designed to mitigate overfitting through sparse feature selection, the fact that validation was performed at a single institution limits generalizability and suggests that further multicenter confirmation is needed. Second, the biological interpretation of radiological features remains partly speculative, as current models often lack spatial resolution and direct mechanistic linkage to underlying gene activity. Radiomic features may reflect general tumor characteristics, such as cellularity or heterogeneity, without directly capturing gene-specific expression patterns.

Future studies incorporating spatial transcriptomics or multiplex immunohistochemistry may help validate these gene-feature associations and define their spatial relationships within tumors. Although the current model may not yet be clinically deployable, it offers a preliminary framework for further exploration of gene-image phenotype associations, feature engineering, and biological interpretability. Prospective multicenter studies with larger and more diverse datasets are needed to refine the model and assess its clinical utility. The inclusion of longitudinal imaging data might improve temporal assessment of prognosis and treatment response. Finally, integrating radiomics with circulating biomarkers such as cfDNA may offer a path to further improve specificity, pending validation in future studies.

## Conclusions

This study suggests the potential of radiogenomics as a noninvasive approach to explore immune-related molecular activity in ccRCC. By integrating quantitative CT-derived radiomic features with transcriptomic data from TCGA, we identified associations between tumor imaging phenotypes and biomarkers linked to NK cell-mediated cytotoxicity, with ICAM1 and RAET1E emerging as preliminary imaging-associated candidate biomarkers. Immunohistochemical validation provided supportive evidence for their differential expression patterns, which correlated with tumor stage and patient survival outcomes, suggesting their potential biological and translational relevance pending further validation. Despite limitations, our findings offer additional insights into the immune landscape of ccRCC and may provide a framework for future prospective studies integrating imaging, genomics, and immunobiology.

## Data Availability

The original contributions presented in the study are included in the article/**Supplementary Material**. Further inquiries can be directed to the corresponding authors.
