# Supplementary material for: CT-based radiogenomic prediction of ICAM1 and RAET1E as biomarkers of NK cytotoxicity in clear cell renal cell carcinoma
Source: Front Immunol. 2026 Jun 3;17:1773251. doi: 10.3389/fimmu.2026.1773251 (PMC13272120; doi:10.3389/fimmu.2026.1773251)
Supplement: Supplementary file 1 [file Table1.docx]

**Table S1 List of radiomics features selected by** *L*_1_**SVM for prediction of ICAM1 expression level**

| **Selected features** | **Radiomic group** | **Filter associated** | **Description** | **D value** |
| --- | --- | --- | --- | --- |
| Root mean squared | First-order | *Wavelet-HLL* | The square-root of the mean of all the squared intensity values. | 4.2131 |
| 90Percentile | First-order | *Wavelet3-HLL* | 90^th^ percentile of the be a set of voxels included in the ROI. | -3.2036 |
| Skewness | First-order | *Wavelet3-HLH* | Measure the asymmetry of the distribution of values about the Mean value. | 2.3832 |
| Median | First-order | *Wavelet-LHL* | The median gray level intensity within the ROI. | -1.7089 |
| Joint energy | GLCM | *Wavelet3-LLL* | A measure of homogeneous patterns in the image. | -1.5394 |
| 90Percentile | First-order | *Wavelet3-LHL* | 90^th^ percentile of the be a set of voxels included in the ROI. | 1.3480 |
| Maximum | First-order | *original* | The maximum gray level intensity within the ROI. | -0.9008 |
| Root mean squared | First-order | *Wavelet3-HHH* | The square-root of the mean of all the squared intensity values. | -0.8024 |
| Root mean squared | First-order | *Wavelet-HHH* | The square-root of the mean of all the squared intensity values. | -0.7822 |
| HGLRE | GLRLM | *Wavelet-LHH* | Measure the distribution of the higher gray-level values, with a higher value indicating a greater concentration of high gray-level values in the image. | 0.7323 |
| SRLGLE | GLRLM | *Wavelet-HHH* | Measure the joint distribution of shorter run lengths with lower gray-level values. | -0.5521 |
| Kurtosis | First-order statistics | *Wavelet-HLL* | A measure of the peakedness of the distribution of values in the image ROI. | -0.5448 |
| Maximum probability | GLCM | *Wavelet3-LHL* | Occurrences of the most predominant pair of neighboring intensity values. | 0.4270 |
| Median | First-order | *Wavelet3-LHL* | The median gray level intensity within the ROI. | 0.3069 |
| Skewness | First-order | *Wavelet3-HLH* | Measure the asymmetry of the distribution of values about the Mean value. | -0.2009 |
| Kurtosis | First-order | *Wavelet2-HLH* | A measure of the peakedness of the distribution of values in the image ROI | -0.0772 |
| LDHGLE | GLDM | *Square* | Measure the joint distribution of large dependence with higher gray-level values. | -0.0659 |

Abbreviations: HGLRE, High Gray Level Run Emphasis; SRLGLE, Short Run Low Gray Level Emphasis; GLRLM, Gray Level Run Length Matrix; GLCM, gray-level co-occurrence matrix; GLDM, Gray Level Dependence Matrix.

**Table S2 List of radiomics features selected by** *L*_1_**SVM for prediction of RAET1E expression level**

| Selected features | Radiomic group | Filter associated | Description | D value |
| --- | --- | --- | --- | --- |
| Gray level variance | GLSZM | *Wavelet2-HLL* | Measure the variance in gray level intensities for the zones. | 0.6818 |
| Dependence variance | GLDM | *Log-sigma-1-5-mm-3D* | Measure the variance in dependence size in the image. | -0.5766 |
| Busyness | NGTDM | *Square* | A measure of the change from a pixel to its neighbour. | -0.4348 |
| Dependence entropy | GLDM | *Wavelet3-LHL* | Measure the entropy in dependence size in the image. | -0.0606 |
| Coarseness | NGTDM | *Coarseness* | A measure of average difference between the center voxel and its neighbourhood and is an indication of the spatial rate of change. | 0.0465 |
| Maximum | First-order | *Wavelet3-LLH* | The maximum gray level intensity within the ROI. | -0.0305 |
| Sum average | GLCM | *Log-sigma-1-mm-3D* | Measure the relationship between occurrences of pairs with lower intensity values and occurrences of pairs with higher intensity values. | -0.0158 |

Abbreviations:GLCM, gray-level co-occurrence matrix; GLDM, Gray Level Dependence Matrix; GLSZM: Gray Level Size Zone Matrix; NGTDM, Neighbouring Gray Tone Difference Matrix.
